# Determinants of neonatal, infant and under-five mortalities: evidence from a developing country, Bangladesh

**DOI:** 10.1057/s41271-023-00413-w

**Published:** 2023-04-28

**Authors:** Md Wahid Murad, A. B. M. Abdullah, Md Mazharul Islam, Md Mahmudul Alam, Carmen Reaiche, Stephen Boyle

**Affiliations:** 1 UniSA Education Futures, University of South Australia, Adelaide, SA Australia; 2 UniSA Business, University of South Australia, 49 North Terrace, Adelaide, SA 5001 Australia; 3 Department of Finance, College of Business, King Abdulaziz University, Jeddah, Saudi Arabia; 4 School of Economics, Finance and Banking, College of Business, Universiti Utara Malaysia, Sintok, Kedah Malaysia; 5 College of Business, Law and Governance, James Cook University, Townsville, QLD Australia

**Keywords:** Neonatal, Infant, Under-five, Mortality, Determinants, Bangladesh, Developing countries

## Abstract

We investigated the macroeconomic determinants of neonatal, infant, and under-five mortalities in Bangladesh for the period 1991–2018 and discuss implications of the United Nations’ Sustainable Development Goal 3 (SDG 3) and Millennium Development Goal 4 (MDG 4) for developing countries. We used annual time series data and the econometric techniques of Fully Modified Ordinary Least Squares (FMOLS) and Dynamic Ordinary Least Squares (DOLS) regressions for analysis. Determinants most effective in combating neonatal, infant, and under-five mortalities include variables such as ‘protecting newborns against tetanus’, ‘increasing healthcare expenditure’, and ‘making sure births are attended by skilled healthcare staff’. Employing more healthcare workers and assuring more and improved healthcare provisions can further reduce the neonatal, infant, and under-five mortalities. Developing countries with similar macroeconomic profiles can achieve similar SDG 3 and MDG 4 outcomes by emulating the policies and strategies Bangladesh applied to reducing child mortalities over the last three decades.

**Supplementary Information:**

The online version contains supplementary material available at 10.1057/s41271-023-00413-w.

## Key messages

- Three most significant macroeconomic determinants of neonatal, infant, and under-five mortalities in Bangladesh are ‘protecting newborns against tetanus’, ‘increasing healthcare expenditure’, and ‘making sure births are attended by skilled healthcare staff’.
- Employing more healthcare workers and assuring more and improved healthcare provisions in relation to the three determinants above could significantly reduce the neonatal, infant, and under-five mortalities in Bangladesh and the other developing countries with similar macroeconomic profiles.
- Developing countries with similar macroeconomic profiles as those of Bangladesh could achieve the SDG 3 and MDG 4 outcomes promptly by emulating the policies and strategies that have been applied to reducing child mortalities in Bangladesh from 1991 to 2018.

## Introduction

**Fig. 1:**
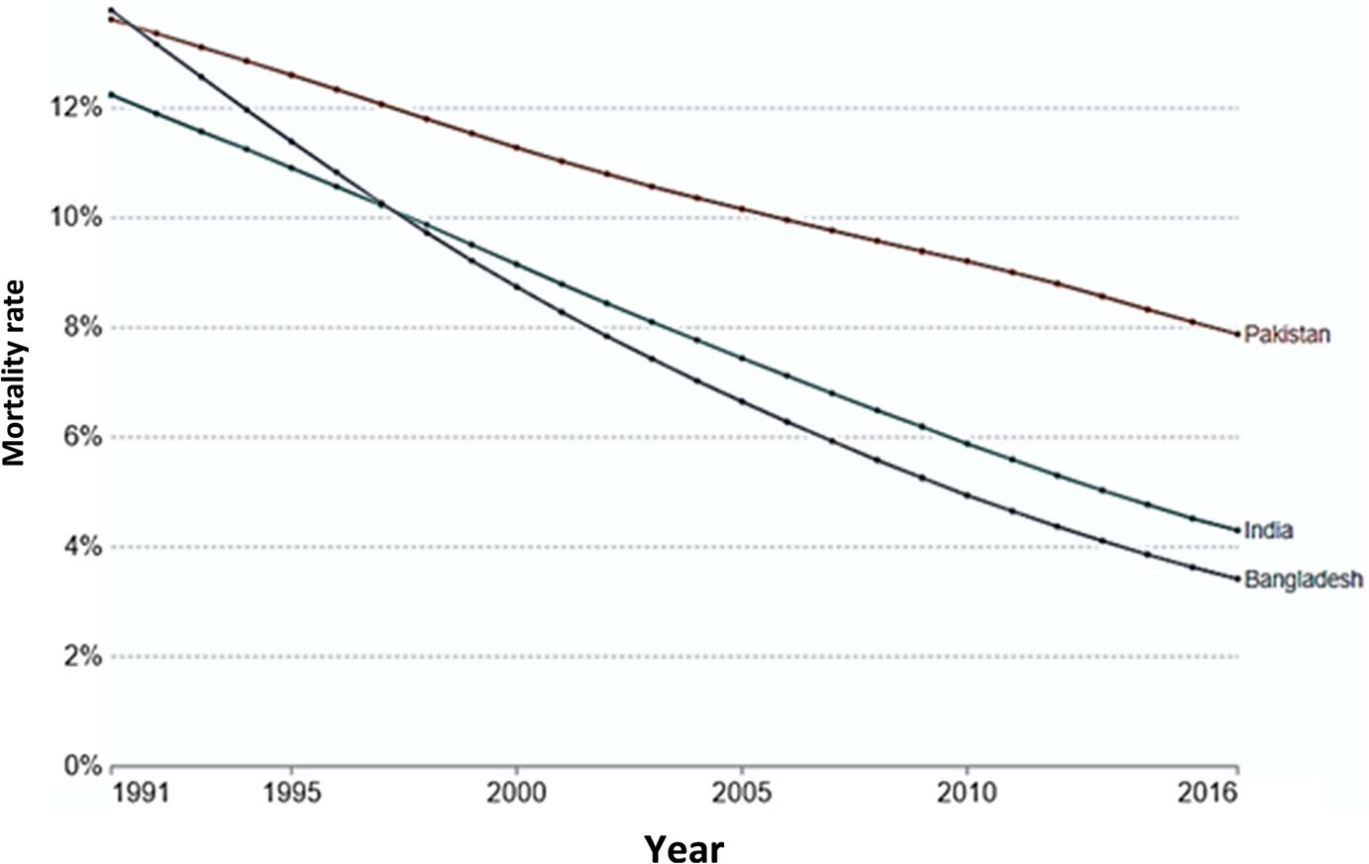
Child mortality in three major Asian nations during 1991–2016 [3, 4]

In its Child Mortality Report 2016, the United Nations Children’s Fund (UNICEF) stated that great progress in reducing child mortality has been made over the past two decades, but more is required to accelerate the progress in reducing child deaths from preventable causes [1]. In 2018, 5.3 million deaths from preventable causes occurred in the first 5 years of life with almost half of these in the 1st month [2]. The United Nations made efforts globally to curtail child mortality [the third Sustainable Development Goal (SDG 3)] and to end preventable deaths of newborns and under-five children by 2030 [the fourth Millennium Development Goal (MDG 4)]. Apparently, child mortality has declined considerably in the developing countries over the last three decades with spectacular reductions reported in some South Asian countries (Fig. 1). Of three major Asian nations Bangladesh outdid India and Pakistan in reducing child mortality and achieving the SDG 3 and MDG 4 targets earlier than targeted.

**Fig. 2:**
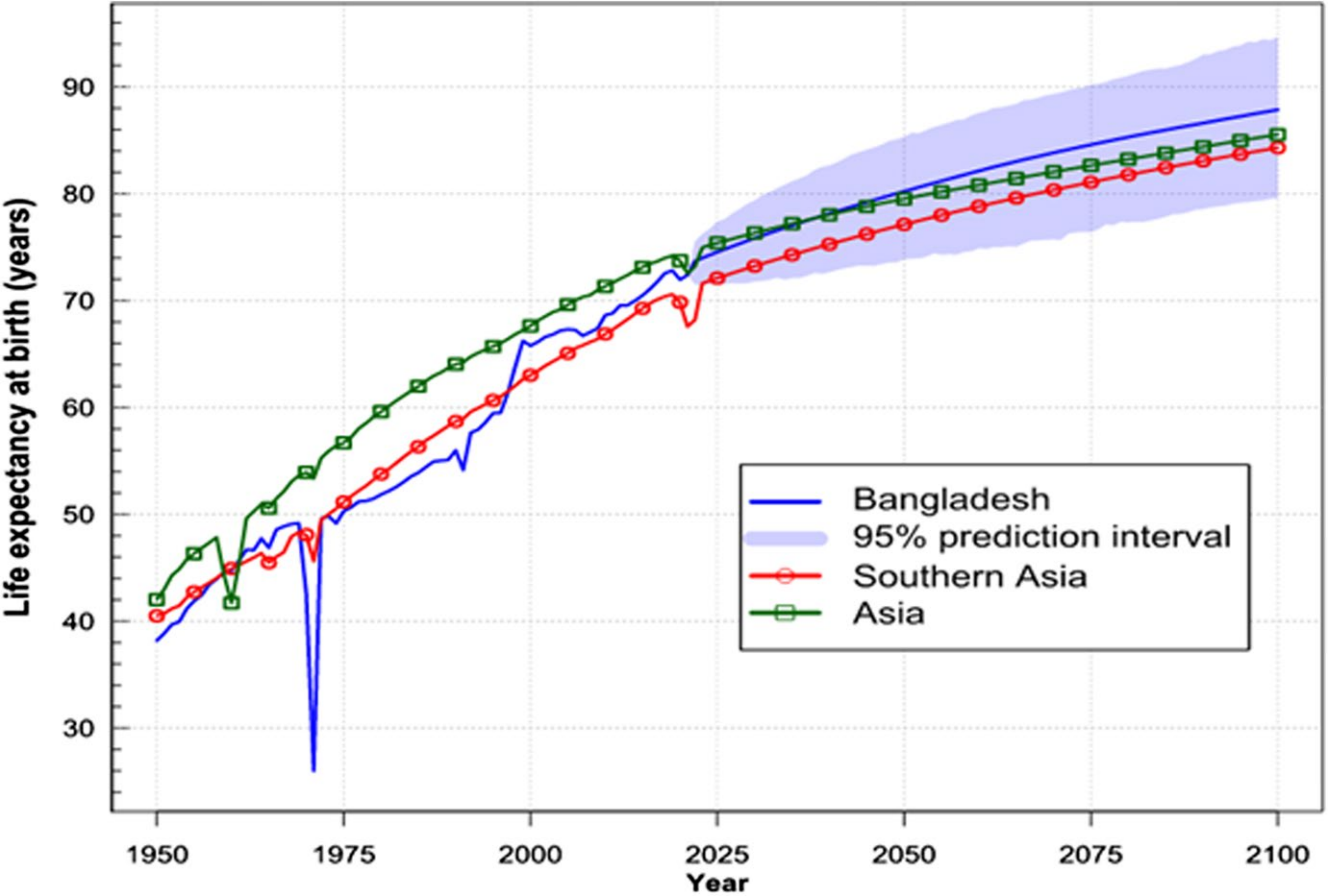
Life expectancy at birth in Bangladesh, South Asia, and Asia [5]

As a highly populated South Asian developing country, Bangladesh demonstrated amazing progress in reducing child mortality. The country transformed its public healthcare system several times since independence from Pakistan in 1971. Currently, it operates with a wide range of healthcare infrastructure and facilities provided by public and private sectors, including government, private sector, non-governmental organizations (NGOs), and donor agencies. Since 1950 the country has greatly improved the health of its people, improved the total life expectancy at birth to 72.05 years, and put the country on track for achieving both the SDG 3 (keeping under-five mortality of 48 deaths per 1000 live births by 2015) and the MDG 4 (reducing child mortality) (Fig. 2).

**Fig. 3:**
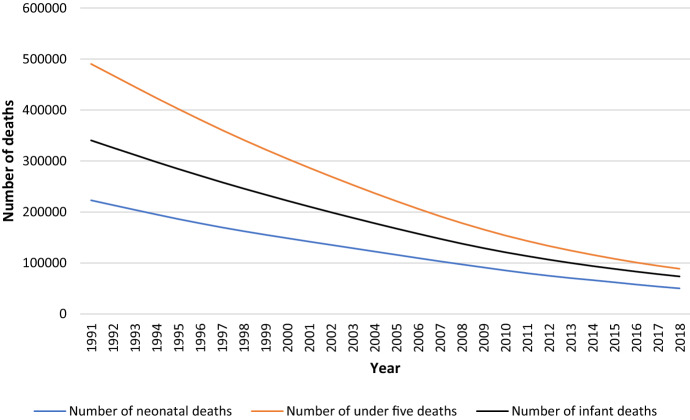
Neonatal, infant and under-five mortality trends in Bangladesh, 1991–2018 (authors produced the figure from World Bank (2019) databank) [7]

Figure 3 below illustrates the neonatal, infant, and under-five child mortality trends in Bangladesh from 1991 to 2018. As the world shifts away from the MDGs era to the SDGs, nations must learn lessons from others where child mortality reductions have been the fastest and design targeted and effective strategies to accelerate reductions in child mortality for the places with the furthest to go [6].

The main objective of this study is to identify and analyze factors that have significantly contributed to such spectacular declines in neonatal, infant, and under-five child mortalities from 1991 to 2018 in Bangladesh. It also discusses the implications of the key findings in relation to the United Nations’ Sustainable Development Goal 3 (SDG 3) and Millennium Development Goal 4 (MDG 4) for other developing countries. The child mortalities involve three measures of deaths: infant deaths, under-five child deaths, and neonatal deaths. Recent studies report that determinants of neonatal, infant, and under-five child mortalities in developing countries are mainly macroeconomic in nature [8–16]. Macro level studies suggest that the role of skilled birth attendant (SBA) on reducing infant mortality should be investigated comprehensively to provide better understanding of what needs to be achieved [17, 18]. Despite existing simple and low-cost interventions, the women and children in Asia and Africa with fewest resources are still deprived of services which can prevent a large number of neonatal deaths [19]. Many developing countries have high infant mortality rates, yet the utilization of SBA services during pregnancy and after birth remains low in these countries [20, 21].

While the mortality of neonatal children often depends on a mother’s state of health, studies have examined the benefits of prenatal care during pregnancy and found it had a significant effect on neonatal mortality [22]. Though most studies have investigated the role of prenatal care in maternal mortality, research has focused less attention on the role of prenatal care on neonatal and infant mortalities [22]. Empirical studies reveal that vaccinations helped to eliminate child mortality [23, 24]. Other studies reported a significant association between timely immunization and child mortality; one found tetanus to be the lead cause of child mortality [25]. A study by Saleh et al. [26] investigated the pre- and post-birth neonatal thrombocytopenia (NNT) in Nigeria and confirmed that childbearing mothers who receive tetanus vaccine experienced less child mortality than those who did not receive it.

Several cross-country studies examined the link between public healthcare expenditure and child mortality and found that healthcare expenditure per capita wielded a significant positive impact on life expectancy and a significant negative impact on infant mortality [27, 28]. Likewise, a study on 25 high-income countries conducted by Erdoğan et al. [29] revealed that healthcare expenditure has a significant and negative effect on infant mortality rate in selected countries. It implies that infant mortality rates of those countries fell as they became more prosperous and rendered public healthcare expenditure more affordable.

Researchers have studied the effects of economic growth and economic contraction on child mortality widely in the contexts of developing countries. Baird et al. [30] found a negative and strong relationship between GDP per capita and infant mortality in Kenya. Pérez-Moreno et al. [31] found that a decrease in GDP per capita led to a significant rise in child mortality rates in the developing countries but found an increase in the same to have had no significant effect on child mortality in those countries.

Some empirical studies show an association between fertility rate and female labor force participation, but a robust relationship between infant mortality and labor force participation remains unexplored [32–34]. Narayan and Smyth [33] argued that there is a potential long term causal relationship between infant mortality and labor force participation. But they argued that the relationship between child mortality and female labor force participation would require further investigation in the context of developing countries.

Developing countries consider population growth as an impediment to economic development because parents of a large family are less able to pay for nutrition, healthcare, and schooling [35]. The World Health Organization’s report by the Commission on Macroeconomics and Health revealed that a low infant mortality rate is strongly associated with low population growth [36]. It is, therefore, important to highlight that increasing growth of population reduces the wellbeing of people in low-income countries and that infant mortality rate shows a dwindling character when predicting population growth [37]. Hence, an in-depth analysis is necessary as part of an investigation into the long-run relationship between infant mortality rates and population growth in the context of developing countries.

## Data and methods

Data we used in the study are annual time-series in nature, extracted from the World Bank Open Data source. Data on neo-natal, infant, and under-five mortalities and other macroeconomic variables covered the period 1991–2018 with no adjustments to the raw data prior to analysis. We conducted the unit root test on the entire data set to determine the appropriate analytical techniques (Supplementary Table S1). As a unit root test produced the first order integration for all the variables with their non-stationary relationships, we chose Fully Modified Ordinary Least Squares (FMOLS) regression and Dynamic Ordinary Least Squares (DOLS) regression as the appropriate analytical techniques to determine the long-run macroeconomic determinants of neonatal, infant, and under-five mortalities. We provide further descriptions of data and analytical techniques in the Supplementary Materials.

## Results and discussion

Estimations obtained through the Fully Modified Ordinary Least Squares (FMOLS) and the Dynamic Ordinary Least Squares (DOLS) regressions produced a negative and highly significant (*p* ≤ 0.01) coefficient for the determinant ‘births attended by skilled healthcare staff’ (Supplementary Table S2). This indicates that assuming all other factors remain constant, an increase in ‘births attended by skilled healthcare staff’ can reduce the child mortality rate, which is consistent under both FMOLS and DOLS estimations. A similar interpretation applies to the relationship between infant mortality and ‘births attended by skilled healthcare staff’ (Supplementary Table S3). These empirical findings are consistent with some earlier studies conducted on other developing countries [17, 38–41]. Skilled healthcare staff members are professionals with sufficient training and skills to manage normal pregnancy childbirth; during the immediate postnatal period they can identify, manage, and refer women and newborns with complications [42]. Several studies further confirm that assistance from skilled healthcare staff during pregnancy and until birth is associated with reduced child and maternal mortalities [17, 38–40].

The variable ‘pregnant women receiving prenatal care’ is conducive to reducing both child mortality rate and infant mortality rate in Bangladesh (Supplementary Tables S2, S3). The FMOLS estimation produced a negative and highly significant (*p* ≤ 0.01) coefficient for the variable, consistent with the finding obtained previously by other studies [22, 43, 44]. Similarly, the variable ‘newborns protected against tetanus’ is highly significant (*p* ≤ 0.01) with a negative coefficient, implying that it is a major factor in reducing both child mortality and infant mortality in Bangladesh (Supplementary Tables S2, S3). This important finding is consistent in both DOLS and FMOLS estimation procedures and is well-supported by recent studies [23–26, 45]. Although the World Health Organization (WHO) and its partners have taken several initiatives to eliminate child mortality caused by not taking tetanus vaccine for NNT, the case fatality rate from this disease remains high and treatment is limited by scarcity of resources and effective drug treatments in developing countries. There has been much progress, however, in improving vaccination coverage, birth hygiene, and surveillance, with specific approaches in high-risk areas. This means that the incidence of the disease continues to decline. We see a steady fall in infant morbidity and mortality in Bangladesh since the establishment of the Expanded Program on Immunization (EPI) in 1979. EPI has been and continues to be effective against fatal diseases such as tuberculosis, diphtheria, tetanus, pertussis, poliomyelitis, and measles in children less than a year old. While the WHO projected national coverage for these vaccines to be over 90% in 2009, the mortality rates remained high at 37 deaths per 1000 live born infants in Bangladesh in 2014 [25].

In the attempt to determine whether ‘healthcare expenditure per capita’ is an important determinant of child mortality and infant mortality in Bangladesh, the empirical finding is highly promising in reducing both child mortality and infant mortality. A negative but a highly significant (*p* ≤ 0.01) coefficient for the variables emerged from both DOLS and FMOLS estimations (Supplementary Tables S2, S3). This very important outcome lends support to the findings of some large-scale and cross-country studies [27–29]. Generally, infant and child deaths can be prevented by providing 16 simple and low-cost interventions [46]. These low-cost strategies should be accessible and available if public health spending rises. Interestingly, when regressed against the numbers of both infant and child deaths as the dependent variables, ‘female labor force participation’ is found to be a positive and highly significant (*p* ≤ 0.01) influence on them in Bangladesh (Supplementary Tables S2, S3). While studies of the relationship between ‘female labor force participation’ and child mortality and infant mortality are quite rare, our empirical findings build on the earlier work of Narayan and Smyth [33]. In both DOLS and FMOLS estimations, ‘population growth’ positively influences both child mortality and infant mortality but is also highly significant (*p* ≤ 0.01) in FMOLS estimation (Supplementary Tables S2, S3). The relationship between ‘population growth’ and child mortality and infant mortality has, so far, been controversial due to varying characteristics of populations and varying levels of access they have to health and other basic amenities and resources across countries. Yet, our finding is consistent with the one found earlier by the World Health Organization [2] and Baker et al. [47].

Again, when regressed against both the dependent variables in DOLS and FMOLS estimations, the ‘GDP growth’ has significantly (*p* ≤ 0.05) reduced both child mortality and infant mortality in Bangladesh (Supplementary Tables S2, S3). As in most countries ‘GDP growth’—if transformed such that it improves the public healthcare system—can reduce both child mortality and infant mortality; this corroborates the findings of some recent studies [1, 27, 30, 31, 48, 49]. Conversely, periods of economic contraction bring additional economic setbacks for the developing economies, leading to worse living conditions for people, particularly children, one of the most vulnerable groups in society. Also, during economic contraction, household income is likely to fall, thus many families have to cut back on expenditure for food and health, and governments often reduce public healthcare spending to control outlays.

This study also investigates whether all the independent variables determine the infant deaths, under-five deaths, and neo-natal deaths in Bangladesh. Accordingly, we have applied both DOLS and FMOLS estimation techniques to determine the relationships among the three dependent variables and the seven independent variables. This strategy helps us to understand which segment of child mortality is best explained by which determinants. Independent variables such as ‘births attended by skilled healthcare staff’, ‘pregnant women receiving prenatal care’, ‘newborns protected against tetanus’, ‘healthcare expenditure per capita’ and ‘GDP growth rate’ all negatively but significantly influenced the infant deaths; both ‘female labor force participation’ and ‘population growth rate’ positively and significantly influence the same (Supplementary Table S4). These findings on Bangladesh are consistent with several studies [1, 27, 30, 31, 48, 49], which have been cited earlier in relation to confirming the significant macroeconomic determinants of infant deaths in other countries.

Similarly, the independent variables ‘births attended by skilled healthcare staff’, ‘pregnant women receiving prenatal care’, ‘newborns protected against tetanus’, ‘healthcare expenditure per capita’ and ‘GDP growth rate’ all negatively but significantly influence the deaths of under-five years old and neonatal children in Bangladesh (Supplementary Tables S5, S6). The other two independent variables, ‘female labor force participation’ and ‘population growth’, influence the under-five child mortality and neonatal death rates both positively and significantly (Supplementary Tables S5, S6). These findings on Bangladesh are consistent with several studies [1, 27, 30, 31, 48, 49], confirming them as the significant macroeconomic determinants of under-five years old child and neonatal child mortalities in other countries. Since 1950, developing countries have implemented many initiatives on lowering the mortalities of under-five years old and neonatal children alongside several initiatives such as promoting economic growth, increasing levels of education, encouraging female empowerment, and provision and acceptance of family planning services. But nevertheless, it is ironic that countries with high child mortality rates have the fastest growing populations in the world. Also, countries with infant mortality rates of less than 20 per 1000 births have an average total fertility rate of 1.7 children, while countries with infant mortality rate of over 100 per 1000 births have an average total fertility rate of 6.2 children [36]. These statistics imply that faster population growth and higher mortality rate in developing countries could be due to resource constraints, as well as a lack of awareness among populations concerning their health and socioeconomic wellbeing.

The strength of empirical findings, as presented and discussed earlier, lies in consistency of the determinants of neonatal, infant, and under-five mortalities identified by both DOLS and FMOLS estimations. In Supplementary Table S7, we show such consistency in terms of the sign of coefficients for independent variables and their level of significance. Except for a few differences in the levels of significance, all the independent variables significantly influence the dependent variables consistently in both estimations. Such consistency of the findings is considered robust, and hence extremely useful for devising and implementing the effective policies on reducing the neonatal, infant, and under-five child mortalities in countries having similar developing and macroeconomic characteristics as Bangladesh. Child mortality is an important indicator of overall health [50]. Appropriate policies centered around its significant macroeconomic determinants could potentially improve the overall health of a nation. Wagner [51] argues that infant mortality is not a health problem but a social problem with health consequences. Thus, any effective solutions for reducing child mortalities would require appropriate social policies aimed at reducing those health consequences among mothers and children.

Because findings from this this study reveal the determinants of neonatal, infant, and under-five mortalities in the context of a developing country, a cross-country panel study across other developing countries would provide a deeper understanding. Inclusion of more countries and use of longitudinal data sets would provide more robust results. Considering the views of health practitioners and policy makers in the analysis would further advance understanding of neonatal, infant, and under-five mortalities and their macroeconomic determinants from both a single country and cross-country perspectives.

## Conclusions

The macroeconomic determinants identified in this study should not be considered exhaustive, because political, economic, social, technological, environmental, and legal elements may also directly or indirectly influence the neonatal, infant and under-five child mortalities in any nation. Coupled with many constraints impinging the public healthcare sector financing and resource acquisition as well as the lack of fundamental health awareness among people, Bangladesh made great strides in reducing the neonatal, infant and under-five child mortalities since 1950. That is why other developing countries can emulate the neonatal, infant and under-five child mortality reduction policies and strategies that Bangladesh applied to reducing the mortalities so significantly over the last three decades. Apparently, Bangladesh applied considerable effort and used its reasonably scarce resources well to achieve the United Nations’ SDG 3 and MDG 4. Yet, the factors that contributed most to its great achievements to date are indeed those macroeconomic efforts to reduce its neonatal, infant and under-five mortalities in the last three decades. Nonetheless, the three most significant strategies that Bangladesh government used in greatly reducing its neonatal, infant, and under-five mortalities included ‘protecting newborns against tetanus’, ‘increasing healthcare expenditure’ and ‘making sure births are attended by skilled healthcare staff’. Thus, we anticipate that allocating more resources to the public healthcare system, providing improved healthcare provisions, and ensuring continuous improvement in the existing services and facilities would help the country to further reduce the child mortalities and to fully achieve the SDG 3 and MDG 4. We expect these empirical findings to have wider applicability in other developing countries, depending on their macroeconomic profiles, and how these are close to or very different from those of Bangladesh.

## Supplementary Information

Below is the link to the electronic supplementary material.

Supplementary file1 (DOCX 48 KB)
