# Supplementary material for: Determinants of neonatal, infant and under-five mortalities: evidence from a developing country, Bangladesh
Source: J Public Health Policy. 2023 Apr 28;44(2):230–41. doi: 10.1057/s41271-023-00413-w (PMC10232642; doi:10.1057/s41271-023-00413-w)
Supplement: Supplementary file 1 — Supplementary file1 (DOCX 48 KB) [file 41271_2023_413_MOESM1_ESM.docx]

**Journal of Public Health Policy**

Supplementary Materials

Determinants of neonatal, infant and under-five mortalities: evidence from a developing country, Bangladesh

Wahid Murad ^1^, ABM Abdullah ^2,*^, Mazharul Islam ^3^, Mahmudul Alam ^4^, Carmen Reaiche ^5^, Stephen Boyle ^5^

1. UniSA Education Futures, University of South Australia, Adelaide, SA, Australia
2. UniSA Business, University of South Australia, Adelaide, SA, Australia
3. Department of Finance, College of Business, King Abdulaziz University, Jeddah, Saudi Arabia
4. School of Economics, Finance and Banking, College of Business, Universiti Utara Malaysia, Sintok, Kedah, Malaysia
5. College of Business, Law and Governance, James Cook University, Townsville, Queensland, Australia

**Corresponding Author:**

ABM Abdullah

[abm.abdullah@unisa.edu.au](mailto:abm.abdullah@unisa.edu.au)

UniSA Business, University of South Australia, 49 North Terrace, Adelaide, SA 5001, Australia

**Running Title:** Determinants of neonatal, infant and under-five mortalities

**Supplementary Data and Methods**

**Data**

The dataset used in the study is extracted from open access databank of the World Bank [1]. The annual data for the period 1991-2018 is used in the estimations. The macroeconomic indicators, which annual data has been extracted on, include infant mortality rate (per 1,000 live births), under-five child mortality rate (per 1,000 live births), number of infant deaths, number of under-five deaths, number of neonatal deaths, births attended by skilled healthcare staff (% of total), newborns protected against tetanus (% of total), pregnant women receiving prenatal care (% of total), public healthcare expenditure per capita (in current US$), female labor force participation (% of total labor force), population growth (annual %), and GDP per capita growth (annual %). All the data is taken in real time.

**Unit root test**

Unit root test on all the time series variables is conducted in order to determine for them the appropriate analytical techniques. For this reason, the Dickey-Fuller (DF) test statistic using a generalized least squares (GLS) rationale is computed for all the variables. All the variables emerged as integrated at I(1) with the assumption of both common and individual unit root process under individual intercept and no trend (Table 1). Noticeably, the application of DF-GLS at both level and I(1) has generated relationships that are non-stationary in nature, thereby producing spurious results. Engle and Granger [2] have contented the direct application of OLS or GLS to non-stationary data produces regressions that are mis-specified or spurious in nature. Such regressions tend to produce performance statistics that are inflated in nature, for example high R^2^ and t-statistics, which often lead investigators to commit very frequent Type I errors [3]. In view of the fact that all variables are integrated at I(1) and OLS or GLS estimator is a biased and inconsistent estimator when applied to non-stationary data, the “group-mean” panel FMOLS estimator developed by Pedroni [4, 5] and the DOLS estimator developed by Stock and Watson [6] were employed to estimate the single cointegrating vector that characterizes the long-run determinants of neonatal, infant and under-five mortalities. The FMOLS estimator not only generates consistent estimates of the *β* parameters in relatively small samples, but it also controls for the likely endogeneity of the regressors and serial correlation [5]. On the other hand, lag and lead terms included in DOLS regression have the purpose of making its stochastic error term independent of all past innovations in stochastic regressors [6].

**Econometric models**

***Fully Modified Ordinary Least Squares (FMOLS) regression***

To achieve the research objectives, this study employs the time series regression techniques of Fully Modified Ordinary Least Squares (FMOLS) and Dynamic Ordinary Least Squares (DOLS). As explained earlier these analytical techniques are considered the most appropriate given the nature of time series data which are being used in the present study. These two analytical techniques are proven robust in characterizing the long-run relationships between the dependent and independent variables. In this study, infant mortality rate (per 1,000 live births), under-five child mortality rate (per 1,000 live births), number of infant deaths, number of under-five deaths, and number of neonatal deaths are measured as the dependent variables. On the other hand, births attended by skilled healthcare staff (% of total), newborns protected against tetanus (% of total), pregnant women receiving prenatal care (% of total), public healthcare expenditure per capita (in current US$), female labor force participation (% of total labor force), population growth (annual %), and GDP per capita growth (annual %) are measured as the independent variables. However, the determinants of neonatal, infant and under-five child mortalities are identified by using both FMOLS and DOLS estimation procedures. Assuming neonatal mortality (NEONATM) as the dependent variable, a prototypical FMOLS equation takes the following form:

${lnNEONATM}_{t}=\beta_{0}+\beta_{1}X_{t}+\varepsilon_{t}$ ……… (1)

Where $\varepsilon_{t}$ is the white noise error$, {lnNEONATM}_{t}$ is the dependent variable, $\beta_{0}$ is the intercept, $\beta_{1}$ is the vector slope coefficient of equation 1 and $X_{t}$is the vector of an independent variable. In our actual FMOLS estimation procedures, the dependent variables that have been regressed against the independent variables are infant mortality rate (per 1,000 live births), under-five child mortality rate (per 1,000 live birth), number of infant deaths, number of under-five deaths, and number of neonatal deaths. That means the child mortality determinants are explained based on these five dependent variables. However, model 1 is estimated by the FMOLS regression due to a few prominent features that it has. This estimation approach was initially applied in a study by Phillips and Hansen [7] to provide optimal estimates of co-integrating regressions. It modifies the Ordinary Least Squares (OLS) to eliminate potential endogeneity problems in the regressors, which are the outcomes of a co-integration relationship among the independent variables. Additionally, the FMOLS approach solves problems caused by the long-run correlation between the co-integrating equation and stochastic regressor changes. The FMOLS estimator, as a time-series analytical technique, is also asymptotically unbiased and characterized as fully efficient in the presence of mixture normal asymptotic allowing for standard Wald tests using asymptotic Chi-square statistical inference. Accordingly, the following linear regression model is considered:

$Y_{t}=\beta_{0}+\acute{\beta_{t}}X_{t}+u_{t}, t=1,2,\ldots\ldots\ldots.n$…………. (2)

Where the vector of I(1) regressors are not cointegrated individually. Therefore, $X_{t}$ has a first-difference stationary process given by:

${\Delta X}_{t}=\vartheta+\nu_{t} where t=2,3,\ldots\ldots..n$…………. (3)

Where $\vartheta$ is a vector of drift parameters and $\nu_{t}$is a vector of I(0), or stationary variables. It is assumed that $\xi_{t}=\left( u_{t},\nu^{'} \right)^{'}$is a strictly stationary process with zero mean and a finite positive-definite covariance matrix,$\Sigma$. However, the computation of FMOLS estimation of $\beta$ is carried out in two stages. In the first stage $Y_{t}$ is corrected for the long-run T interdependence of $u_{t}$ and $\nu_{t}$. For this purpose, $\left( u_{t},\nu^{'} \right)^{'}$ is the OLS residual vector in equation (2) and it takes the below form:

$\xi_{t}=\left( \begin{matrix} \hat{u}_{t} \\ \hat{\nu}_{t} \end{matrix} \right), t=2,3\ldots.n$…………. (4)

Where $\hat{\nu}_{t}=\Delta X_{t}-\hat{\mu} for t=2,3,\ldots,n$ and $\hat{\mu}_{t}=\left( n-1 \right)^{-1}\sum_{t=2}^{n} \Delta X_{t}.$ A consistent estimator of the long-run variance of $\xi_{t}$is given by:

$\hat{\Omega}=\hat{\Sigma}+\hat{\Lambda}+\Lambda^{'}=\left[ \begin{matrix} \begin{matrix} \hat{\Omega}_{11} \\ 1 \end{matrix}x1^{\hat{\Omega}_{11}} & \begin{matrix} \hat{\Omega}_{21} \\ 1 \end{matrix}xk^{\hat{\Omega}_{21}} \\ \begin{matrix} \hat{\Omega}_{21} \\ k \end{matrix}x1^{\hat{\Omega}_{21}} & \begin{matrix} \hat{\Omega}_{22} \\ 1 \end{matrix}x1^{\hat{\Omega}_{22}} \end{matrix} \right]$…………. (5)

Where $\hat{\Sigma}=\frac{1}{n-1}\sum_{t=2}^{n} \hat{\xi}_{t}\hat{\xi}_{t},\hat{\Lambda}=\sum_{s=1}^{m} w(s,m)\hat{\Gamma}_{s},\hat{\Gamma}_{s}=n^{-1}\sum_{t=1}^{n-s} \hat{\xi}_{t}{\hat{\xi}^{'}}_{t+s}$ and $w(s,m)$is the lag window with horizon *m*. Now let us state some conditions of the FMOLS estimation approach:

$\hat{\Delta}=\hat{\Sigma}=\hat{\Lambda}=\left[ \begin{matrix} \hat{\Lambda}_{11} & \hat{\Lambda}_{12} \\ \hat{\Lambda}_{21} & \hat{\Lambda}_{22} \end{matrix} \right]$…………. (6)

$\hat{Z}=\hat{\Delta}_{21}-\hat{\Delta}_{22}{\hat{\Omega_{22}}}^{-1}\hat{\Omega}_{21}$…………. (7)

$\hat{Z}=\hat{\Delta}_{21}-\hat{\Delta}_{22}{\hat{\Omega_{22}}}^{-1}\hat{\Omega}_{21}$…………. (8)

${\hat{Y}^{*}}_{t}=Y_{t}-\hat{\Omega}_{12}{\hat{\Omega_{t}}}^{-1}\hat{\upsilon_{t}}$…………. (9)$\left( k+1 \right) x k=\left[ \begin{matrix} \begin{matrix} 0 \\ 1 \end{matrix}xk^{0} \\ \begin{matrix} lk \\ k \end{matrix}xk^{lk} \end{matrix} \right]$…………. (10)

In the second stage, the FMOLS estimator of $\beta$ is given by:

$\hat{\beta}^{*}=\left( W^{'}W \right)^{-1}(W^{'}\hat{Y}^{*}-nD\hat{Z}$), …………. (11)

Where $\hat{Y}^{*}=\left( {\hat{Y}^{*}}_{1}, {\hat{Y}^{*}}_{2},\ldots\ldots{\hat{Y}^{*}}_{n} \right)^{'}$, $W=\left( \tau_{n},X \right), and \tau_{n}={(1,1,1\ldots1)}^{'}$.

***Dynamic Ordinary Least Squares (DOLS) regression***

As mentioned earlier the technique of DOLS is employed as an alternative single equation estimation procedure. The key benefit of using DOLS approach is that it takes into consideration the presence of a mixed order of integration of the respective variables in the co-integrated framework. The estimation of DOLS involves regressing one of the I(1) variable against other I(1) and I(0) variables by taking leads (p) and lags (-p) in the framework [8]. Consequently, this single equation estimator solves possible endogenous bias and small sample bias issues. The estimated co-integrating vectors from DOLS estimators are asymptotically efficient. Stock and Watson [6] provide evidence, based on Monte Carlo experiments, that the DOLS estimators perform well relative to the other asymptotically efficient estimators. With χ^2^ inference its most robust ability is the accommodability of higher order of integration of time series data. A prototypical form of Stock-Watson’s DOLS model, which is employed to identify the macroeconomic determinants of neonatal, infant and under-five mortalities, is presented below:

$Y_{t}=\beta_{o}+\vec{\beta}X+\sum_{j=-q}^{p} \vec{d}_{j}\Delta X_{t-j}+u_{t}$…………. (12)

Where *Y_t_* is dependent variable, *X* is matrix of explanatory variables, $\vec{\beta}$ is cointegrating vector representing the long-run effect of a change in X on Y, *p* is lag length, *q* is lead length and *u_t_* is error term.

**Supplementary Table S1.** Unit root test results of DF-GLS tests.

| Variables | Level | 1^st^ Difference |
| --- | --- | --- |
| Infant mortality rate (per 1,000 live births) | 0.240 | 3.837 |
| Number of infant deaths | 1.450 | -3.407 |
| Number of under-five deaths | -2.059 | -3.517 |
| Number of neonatal deaths | -2.491 | -2.980 |
| Under-five mortality rate (per 1,000 live births) | -2.884 | -3.509 |
| Births attended by skilled healthcare staff (% of total) | -1.083 | -6.128 |
| Pregnant women receiving prenatal care (% of total) | -1.885 | -3.665 |
| Newborns protected against tetanus (% of total) | -1.207 | -5.120 |
| Healthcare expenditure per capita (in current US$) | -1.318 | -3.517 |
| Female labor force participation (% of total labor force) | -0.955 | -3.803 |
| Population growth rate | -1.186 | -4.925 |
| GDP growth rate | -2.627 | -3.021 |

**Supplementary Table S2.** Macroeconomic determinants of child mortality (mortality rate of per 1,000 children under-five years old as the dependent variable).

| Variables | DOLS | FMOLS |
| --- | --- | --- |
| Births attended by skilled healthcare staff (% of total) | -0.265^***^  (0.0964) | -0.280^***^  (0.0231) |
|  |  |  |
| Pregnant women receiving prenatal care (%) | -0.178  (0.132) | -0.244^***^  (0.0353) |
|  |  |  |
| Newborns protected against tetanus (%) | -0.682^***^  (0.254) | -0.803^***^  (0.0676) |
|  |  |  |
| Healthcare expenditure per capita (in current US$) | -0.396^***^  (0.140) | -0.292^***^  (0.0409) |
|  |  |  |
| Female labor force participation (% of total labor force) | 3.957^***^  (1.489) | 2.540^***^  (0.489) |
|  |  |  |
| Population growth rate | 0.101  (0.0901) | 0.0720^***^  (0.0227) |
|  |  |  |
| GDP growth rate | -0.0124  (0.0141) | -0.00696^*^  (0.00359) |
|  |  |  |
| Constant | -4.852  (6.333) | 0.919  (2.042) |
|  |  |  |
| Observations | 25 | 24 |
| R-squared | 0.993 | 0.992 |

Notes:

1. Standard errors of the coefficients are in parentheses
2. *Significant at the 10% level (p<0.1)
3. ***Significant at the 1% level (p<0.01)

**Supplementary Table S3.** Macroeconomic determinants of infant mortality (infant mortality rate per 1,000 live births as the dependent variable).

| Variables | DOLS | FMOLS |
| --- | --- | --- |
| Births attended by skilled healthcare staff (% of total) | -0.234^***^  (0.0862) | -0.247^***^  (0.0206) |
|  |  |  |
| Pregnant women receiving prenatal care (%) | -0.150  (0.118) | -0.210^***^  (0.0314) |
|  |  |  |
| Newborns protected against tetanus (%) | -0.602^***^  (0.227) | -0.712^***^  (0.0601) |
|  |  |  |
| Healthcare expenditure per capita | -0.357^***^  (0.125) | -0.263^***^  (0.0364) |
|  |  |  |
| Female labor force participation (% of total labor force) | 3.646^***^  (1.332) | 2.360^***^  (0.435) |
|  |  |  |
| Population growth rate | 0.0824  (0.0806) | 0.0559^***^  (0.0202) |
|  |  |  |
| GDP growth rate | -0.0107  (0.0126) | -0.00577^*^  (0.00319) |
|  |  |  |
| Constant | -4.619  (5.665) | 0.616  (1.818) |
|  |  |  |
| Observations | 25 | 24 |
| R-squared | 0.992 | 0.992 |

Notes:

1. Standard errors of the coefficients are in parentheses
2. *Significant at the 10% level (p<0.1)
3. ***Significant at the 1% level (p<0.01)

**Supplementary Table S4.** Macroeconomic determinants of infant deaths (number of infant deaths as the dependent variable).

| Variables | DOLS | FMOLS |
| --- | --- | --- |
|  |  |  |
| Births attended by skilled healthcare staff (% of total) | -0.227^**^  (0.0940) | -0.240^***^  (0.0227) |
|  |  |  |
| Pregnant women receiving prenatal care (%) | -0.0592  (0.129) | -0.116^***^  (0.0347) |
|  |  |  |
| Newborns protected against tetanus (%) | -0.654^***^  (0.248) | -0.755^***^  (0.0664) |
|  |  |  |
| Healthcare expenditure per capita (in current US$) | -0.452^***^  (0.136) | -0.361^***^  (0.0402) |
|  |  |  |
| Female labor force participation (% of total labor force) | 3.584^**^  (1.452) | 2.361^***^  (0.480) |
|  |  |  |
| Population growth rate | 0.187^**^  (0.0879) | 0.163^***^  (0.0223) |
|  |  |  |
| GDP growth rate | -0.01000  (0.0137) | -0.00518  (0.00353) |
|  |  |  |
| Constant | 3.693  (6.176) | 8.651^***^  (2.008) |
|  |  |  |
| Observations | 25 | 24 |
| R-squared | 0.993 | 0.993 |

Notes:

1. Standard errors of the coefficients are in parentheses
2. **Significant at the 5% level (p< 0.05)
3. ***Significant at the 1% level (p<0.01)

**Supplementary Table S5.** Macroeconomic determinants of under-five years old child deaths (number of under five deaths as the dependent variable).

| Variables | DOLS | FMOLS |
| --- | --- | --- |
| Births attended by skilled healthcare staff (% of total) | -0.271^**^  (0.107) | -0.285^***^  (0.0257) |
|  |  |  |
| Pregnant women receiving prenatal care (%) | -0.0993  (0.146) | -0.163^***^  (0.0393) |
|  |  |  |
| Newborns protected against tetanus (%) | -0.751^***^  (0.281) | -0.867^***^  (0.0752) |
|  |  |  |
| Healthcare expenditure per capita (in current US$) | -0.494^***^  (0.154) | -0.391^***^  (0.0455) |
|  |  |  |
| Labor force participation by female (% of total labor force) | 4.033^**^  (1.645) | 2.645^***^  (0.544) |
|  |  |  |
| Population growth rate | 0.179^*^  (0.0996) | 0.151^***^  (0.0252) |
|  |  |  |
| GDP growth rate | -0.0126  (0.0156) | -0.00712^*^  (0.00399) |
|  |  |  |
| Constant | 3.174  (6.999) | 8.807^***^  (2.272) |
|  |  |  |
| Observations | 25 | 24 |
| R-squared | 0.993 | 0.993 |

Notes:

1. Standard errors of the coefficients are in parentheses
2. *Significant at the 10% level (p<0.1)
3. **Significant at the 5% level (p< 0.05)
4. ***Significant at the 1% level (p<0.01)

**Supplementary Table S6.** Macroeconomic determinants of neonatal deaths (number of neonatal deaths as the dependent variable)

| Variables | DOLS | FMOLS |
| --- | --- | --- |
|  |  |  |
| Births attended by skilled healthcare staff (% of total) | -0.186^**^  (0.0842) | -0.200^***^  (0.0198) |
|  |  |  |
| Pregnant women receiving prenatal care (%) | -0.0239  (0.116) | -0.0873^***^  (0.0302) |
|  |  |  |
| Newborns protected against tetanus (%) | -0.617^***^  (0.222) | -0.732***  (0.0579) |
|  |  |  |
| Healthcare expenditure per capita (in current US$) | -0.429^***^  (0.122) | -0.329^***^  (0.0351) |
|  |  |  |
| Labor force participation by female (% of total labor force) | 3.547^***^  (1.301) | 2.188^***^  (0.419) |
|  |  |  |
| Population growth rate | 0.145^*^  (0.0788) | 0.117^***^  (0.0194) |
|  |  |  |
| GDP growth rate | -0.0105  (0.0123) | -0.00517^*^  (0.00307) |
|  |  |  |
| Constant | 3.065  (5.534) | 8.593^***^  (1.751) |
|  |  |  |
| Observations | 25 | 24 |
| R-squared | 0.992 | 0.993 |

Notes:

1. Standard errors of the coefficients are in parentheses
2. *Significant at the 10% level (p<0.1)
3. **Significant at the 5% level (p< 0.05)
4. ***Significant at the 1% level (p<0.01)

**Supplementary Table S7.** Macroeconomic determinants of neonatal, infant and under-five child mortalities in Bangladesh.

| DOLS  FMOLS | | Independent variables | | | | | | |
| --- | --- | --- | --- | --- | --- | --- | --- | --- |
|  |  | Births attended by skilled healthcare staff | Pregnant women receiving prenatal care | Newborns protected against tetanus | Healthcare expenditure per capita | Female labor force participation | Population growth rate | GDP growth rate |
| Dependent variables | Under-five mortality rate | +ve  p≤0.01 | -ve  NS | -ve  p≤0.01 | -ve  p≤0.01 | +ve  p≤0.01 | +ve  NS | -ve  NS |
|  |  | +ve  p≤0.01 | -ve  p≤0.01 | -ve  p≤0.01 | -ve  p≤0.01 | +ve  p≤0.01 | +ve  p≤0.01 | -ve  p≤0.1 |
|  | Infant mortality rate | -ve  p≤0.01 | -ve  NS | -ve  p≤0.01 | -ve  p≤0.01 | +ve  p≤0.01 | +ve  NS | -ve  NS |
|  |  | -ve  p≤0.01 | -ve  p≤0.01 | -ve  p≤0.01 | -ve  p≤0.01 | +ve  p≤0.01 | +ve  p≤0.01 | +ve  p≤0.1 |
|  | Number of infant deaths | -ve  p≤0.01 | -ve  NS | -ve  p≤0.01 | -ve  p≤0.01 | +ve  p≤0.01 | +ve  p≤0.05 | -ve  NS |
|  |  | -ve  p≤0.01 | -ve  p≤0.01 | -ve  p≤0.01 | -ve  p≤0.01 | +ve  p≤0.01 | +ve  p≤0.01 | -ve  NS |
|  | Number of under five deaths | -ve  p≤0.05 | -ve  NS | -ve  p≤0.01 | -ve  p≤0.01 | +ve  p≤0.01 | +ve  p≤0.1 | -ve  NS |
|  |  | -ve  p≤0.01 | -ve  p≤0.01 | -ve  p≤0.01 | -ve  p≤0.01 | +ve  p≤0.01 | +ve  p≤0.01 | -ve  p≤0.1 |
|  | Number of neonatal deaths | -ve  p≤0.05 | -ve  NS | -ve  p≤0.01 | -ve  p≤0.01 | +ve  p≤0.01 | +ve  p≤0.1 | -ve  NS |
|  |  | -ve  p≤0.01 | -ve  p≤0.01 | -ve  p≤0.01 | -ve  p≤0.01 | +ve  p≤0.01 | +ve  p≤0.01 | -ve  p≤0.1 |

Notes:

1. “p<0.1” indicates significant at the 10% level
2. “p< 0.05” indicates significant at the 5% level
3. “p<0.01” indicates significant at the 1% level
4. “NS” indicates not significant

**Supplementary References**

1. World Bank. World Bank Databank: Free and open access to global development data. WB 2019. <https://data.worldbank.org>, Accessed 25 Sep 2021.
2. Engle RF, Granger CWJ. Cointegration and error correction: representation, estimation, and testing. Econometrica. 1987; 55(2): 251-76.
3. Granger CWJ, Newbold P. Spurious regression in econometrics. Journal of Econometrics. 1974; 2(2): 111-120.
4. Pedroni P. Critical values for cointegration tests in heterogeneous panels with multiple regressors. Oxford Bulletin of Economics and Statistics (Special Issue). 1999a; 61:653-670.
5. Pedroni P. Fully Modified OLS for Heterogeneous Cointegrated Panels. Working Paper, Indiana University, December. 1999b; pp. 1-40.
6. Stock JH, Watson MW. A simple estimator of cointegrating vectors in higher order integrated systems. Econometrica. 1993; 61(4): 783-820.
7. Phillips PC, Hansen BE. Statistical inference in instrumental variables regression with I(1) processes. Review of Economic Studies. 1990; 57(1): 99–125.
8. Ang JB. Financial reforms, patent protection, and knowledge accumulation in India. World Development. 2010; 38(8):1070–81.
